# *meso*-5,5′-Bis[(4-fluoro­phen­yl)diazen­yl]-2,2′-(pentane-3,3-di­yl)di-1*H*-pyrrole

**DOI:** 10.1107/S1600536810053535

**Published:** 2011-01-08

**Authors:** Boyang Li, Guilong Zhang, Shipeng Sun, Zhenming Yin

**Affiliations:** a Tianjin Key Laboratory of Structure and Performance for Functional Molecules, College of Chemistry, Tianjin Normal Uinversity, Tianjin 300387, People’s Republic of China; b Agro-Environmental Protection Institute, Ministry of Agriculture, Tianjin 300191, People’s Republic of China

## Abstract

There are two independent molecules in the asymmetric unit of the title compound, C_25_H_24_F_2_N_6_, in which the N=N bonds adopt a *trans* configuration with distances in the range 1.262 (2)–1.269 (3) Å. The dihedral angles between heterocycles are 86.7 (2) and 85.6 (2)° in the two molecules while the dihedral angles between the heterocylic rings and the adjacent benzene rings are 13.4 (2) and 13.4 (2)° in one molecule and 5.3 (2) and  6.5 (2)° in the other. In the crystal, pairs of independent mol­ecules are held together by four N—H⋯N hydrogen bonds, forming inter­locked dimers.

## Related literature

For the crystal structrues of chloro-, bromo- and iodo- substituted 5,5′-bis­phenyl­diazo-dipyrromethane, see: Yin *et al.* (2009 ▶). For halogen bonding, see: Metrangolo *et al.* (2008 ▶).
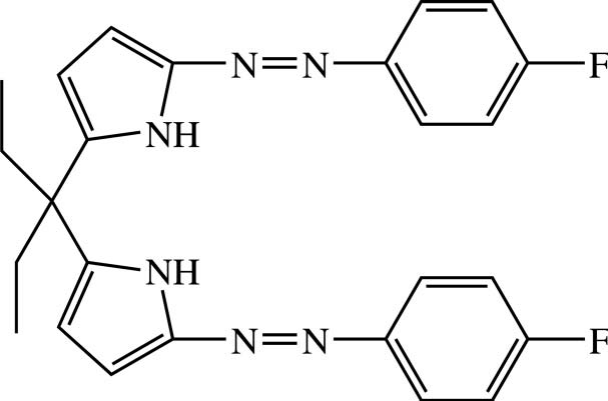


## Experimental

### 

#### Crystal data

C_25_H_24_F_2_N_6_
*M* *_r_* = 446.50
Monoclinic, 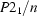

*a* = 9.727 (2) Å
*b* = 29.896 (7) Å
*c* = 16.366 (4) Å
β = 90.745 (4)°
*V* = 4758.9 (19) Å^3^
*Z* = 8
Mo *K*α radiation
μ = 0.09 mm^−1^
*T* = 296 K
0.38 × 0.30 × 0.22 mm

#### Data collection

Bruker SMART CCD area-detector diffractometer
Absorption correction: multi-scan (*SADABS*; Bruker, 1997 ▶) *T*_min_ = 0.967, *T*_max_ = 0.981
24394 measured reflections
8418 independent reflections
5462 reflections with *I* > 2σ(*I*)
*R*_int_ = 0.025

#### Refinement

*R*[*F* ^2^ > 2σ(*F* ^2^)] = 0.048
*wR*(*F* ^2^) = 0.140
*S* = 1.01
8418 reflections
599 parameters
H-atom parameters constrained
Δρ_max_ = 0.23 e Å^−3^
Δρ_min_ = −0.30 e Å^−3^

### 

Data collection: *SMART* (Bruker, 1997 ▶); cell refinement: *SAINT* (Bruker, 1997 ▶); data reduction: *SAINT*; program(s) used to solve structure: *SHELXS97* (Sheldrick, 2008 ▶); program(s) used to refine structure: *SHELXL97* (Sheldrick, 2008 ▶); molecular graphics: *SHELXTL* (Sheldrick, 2008 ▶); software used to prepare material for publication: *SHELXTL*.

## Supplementary Material

Crystal structure: contains datablocks global, I. DOI: 10.1107/S1600536810053535/jh2246sup1.cif

Structure factors: contains datablocks I. DOI: 10.1107/S1600536810053535/jh2246Isup2.hkl

Additional supplementary materials:  crystallographic information; 3D view; checkCIF report

## Figures and Tables

**Table 1:** Hydrogen-bond geometry (Å, °)

*D*—H⋯*A*	*D*—H	H⋯*A*	*D*⋯*A*	*D*—H⋯*A*
N3—H3⋯N12	0.86	2.27	3.121 (3)	171
N4—H4⋯N7	0.86	2.22	3.062 (2)	166
N9—H9⋯N6	0.86	2.31	3.160 (3)	168
N10—H10⋯N1	0.86	2.26	3.092 (3)	163

**Fig. 1.**
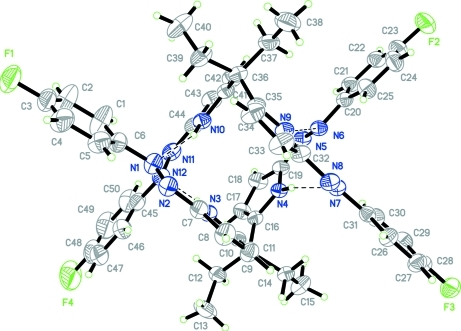
The molecular structure of (I), with the atom-numbering scheme and 30% probability displacement ellipsoids.

## supplementary crystallographic information

### Comment

In previous work, we reported the crystal structrues of chloro, bromo and iodo substituted 5,5'-bisphenyldiazo-dipyrromethane compounds (Yin *et al.* 2009). Herein, we report the crystal of 5,5'-bis(4-fluorophenyldiazo) -dipyrromethane (I). The molecular structrue of (I) is shown in Fig. 1. In the structrue, all the N ═N adopt *trans* conformantion and their distance is at the range of 1.262 (2)Å to 1.269 (3) Å, which is shorter than that of its analogues. Same to its analogues, two molecules of the title compound are held together by four N—H···N hydrogen bonds and consequently form an interlocked type dimer. However, in the crystal structrue of (I), no halogen···π interaction is observed. It indicates that the fluorine atom is a poor halogen bonding donor (Metrangolo *et al.* 2008).

### Experimental

A 0°C solution of the 4-fluoroaniline (5 mmol) and aqueous HCl (4 ml) in water 4 (ml) was treated with a 0°C solution of NaNO_2_ (0.35 g, 5 mmol) in water (10 ml), and the mixture was stirred at 0°C for 0.5 h. The diazonium salt solution was added drop wise to the solution of dipyrromethane (0.5 g, 2.5 mmol) in acetonitrile (25 ml) and three drops of acetic acid. The combined solution was maintained at 0°C for 2 h with stirring. After this time, EtOAc (25 ml) and water (25 ml) were added. The organic layer was separated and washed with water (20 ml) and dried with anhydrous MgSO_4_. The dried solution was evaporated and the residue was purified by column chromatography on silica.

### Refinement

N—H located from difference map and refined freely. Other H atoms were placed in difference Fourier map (C—H = 0.93 or 0.97 Å) and included in the final cycles of refinement using a riding model, with *U*_iso_(H) = 1.2 *U*_eq_(C).

### Crystal data

**Table:**

C_25_H_24_F_2_N_6_	*D*_x_ = 1.246 Mg m^−^^3^
*M**_r_* = 446.50	Melting point: 438 K
Monoclinic, *P*2_1_/*n*	Mo *K*α radiation, λ = 0.71073 Å
*a* = 9.727 (2) Å	Cell parameters from 5736 reflections
*b* = 29.896 (7) Å	θ = 2.5–23.5°
*c* = 16.366 (4) Å	µ = 0.09 mm^−^^1^
β = 90.745 (4)°	*T* = 296 K
*V* = 4758.9 (19) Å^3^	Block, red
*Z* = 8	0.38 × 0.30 × 0.22 mm
*F*(000) = 1872	

### Data collection

**Table:**

Bruker SMART CCD area-detector diffractometer	8418 independent reflections
Radiation source: fine-focus sealed tube	5462 reflections with *I* > 2σ(*I*)
graphite	*R*_int_ = 0.025
φ and ω scans	θ_max_ = 25.0°, θ_min_ = 1.4°
Absorption correction: multi-scan (*SADABS*; Bruker, 1997)	*h* = −7→11
*T*_min_ = 0.967, *T*_max_ = 0.981	*k* = −33→35
24394 measured reflections	*l* = −19→18

### Refinement

**Table:**

Refinement on *F*^2^	Primary atom site location: structure-invariant direct methods
Least-squares matrix: full	Secondary atom site location: difference Fourier map
*R*[*F*^2^ > 2σ(*F*^2^)] = 0.048	Hydrogen site location: inferred from neighbouring sites
*wR*(*F*^2^) = 0.140	H-atom parameters constrained
*S* = 1.01	*w* = 1/[σ^2^(*F*_o_^2^) + (0.0568*P*)^2^ + 1.4249*P*] where *P* = (*F*_o_^2^ + 2*F*_c_^2^)/3
8418 reflections	(Δ/σ)_max_ < 0.001
599 parameters	Δρ_max_ = 0.23 e Å^−^^3^
0 restraints	Δρ_min_ = −0.30 e Å^−^^3^

### Special details

**Table:**

Geometry. All e.s.d.'s (except the e.s.d. in the dihedral angle between two l.s. planes) are estimated using the full covariance matrix. The cell e.s.d.'s are taken into account individually in the estimation of e.s.d.'s in distances, angles and torsion angles; correlations between e.s.d.'s in cell parameters are only used when they are defined by crystal symmetry. An approximate (isotropic) treatment of cell e.s.d.'s is used for estimating e.s.d.'s involving l.s. planes.
Refinement. Refinement of *F*^2^ against ALL reflections. The weighted *R*-factor *wR* and goodness of fit *S* are based on *F*^2^, conventional *R*-factors *R* are based on *F*, with *F* set to zero for negative *F*^2^. The threshold expression of *F*^2^ > σ(*F*^2^) is used only for calculating *R*-factors(gt) *etc*. and is not relevant to the choice of reflections for refinement. *R*-factors based on *F*^2^ are statistically about twice as large as those based on *F*, and *R*- factors based on ALL data will be even larger.

### Fractional atomic coordinates and isotropic or equivalent isotropic displacement parameters (Å^2^)

**Table:**

	*x*	*y*	*z*	*U*_iso_*/*U*_eq_	
F1	−0.0313 (2)	0.40754 (6)	0.99124 (13)	0.1342 (7)	
F2	−0.0356 (2)	0.05104 (6)	0.32465 (10)	0.1261 (7)	
F3	0.62392 (17)	−0.08458 (5)	0.69508 (10)	0.0940 (5)	
F4	−0.1461 (4)	0.13976 (9)	1.30129 (14)	0.2276 (17)	
N1	0.1615 (2)	0.23831 (6)	0.92959 (13)	0.0696 (5)	
N2	0.2903 (2)	0.23202 (6)	0.93140 (12)	0.0667 (5)	
N3	0.24026 (17)	0.15258 (6)	0.93361 (10)	0.0559 (4)	
H3	0.1526	0.1545	0.9391	0.067*	
N4	0.11790 (15)	0.06841 (5)	0.81295 (10)	0.0505 (4)	
H4	0.1871	0.0766	0.7844	0.061*	
N5	−0.04556 (17)	0.05686 (6)	0.70124 (11)	0.0579 (5)	
N6	0.04364 (17)	0.07351 (6)	0.65450 (11)	0.0570 (4)	
N7	0.37178 (17)	0.07958 (6)	0.70618 (10)	0.0560 (4)	
N8	0.44494 (18)	0.11432 (6)	0.70152 (11)	0.0608 (5)	
N9	0.23127 (16)	0.15696 (6)	0.69593 (10)	0.0550 (4)	
H9	0.1750	0.1349	0.6917	0.066*	
N10	−0.03734 (17)	0.18636 (6)	0.81523 (11)	0.0586 (5)	
H10	0.0308	0.1965	0.8436	0.070*	
N11	−0.17156 (19)	0.15596 (7)	0.92686 (14)	0.0713 (5)	
N12	−0.0703 (2)	0.16435 (7)	0.97406 (13)	0.0687 (5)	
C1	−0.0185 (3)	0.29074 (10)	0.9473 (3)	0.1207 (13)	
H1	−0.0789	0.2670	0.9389	0.145*	
C2	−0.0702 (4)	0.33242 (12)	0.9631 (3)	0.1312 (14)	
H2	−0.1646	0.3371	0.9654	0.157*	
C3	0.0176 (4)	0.36597 (10)	0.97501 (19)	0.0934 (9)	
C4	0.1538 (4)	0.36035 (9)	0.9703 (2)	0.1035 (10)	
H4A	0.2126	0.3846	0.9780	0.124*	
C5	0.2072 (3)	0.31833 (9)	0.95400 (18)	0.0855 (8)	
H5	0.3017	0.3143	0.9502	0.103*	
C6	0.1204 (2)	0.28307 (8)	0.94356 (15)	0.0670 (6)	
C7	0.3277 (2)	0.18809 (8)	0.92675 (14)	0.0610 (6)	
C8	0.4581 (2)	0.17116 (9)	0.91794 (16)	0.0759 (7)	
H8	0.5382	0.1878	0.9120	0.091*	
C9	0.4486 (2)	0.12509 (8)	0.91942 (15)	0.0689 (6)	
H9A	0.5214	0.1052	0.9140	0.083*	
C10	0.3129 (2)	0.11375 (7)	0.93039 (12)	0.0541 (5)	
C11	0.2467 (2)	0.06931 (7)	0.94670 (12)	0.0533 (5)	
C12	0.2067 (2)	0.06721 (9)	1.03769 (13)	0.0701 (6)	
H12A	0.1365	0.0895	1.0474	0.084*	
H12B	0.1664	0.0381	1.0483	0.084*	
C13	0.3235 (3)	0.07462 (13)	1.09824 (17)	0.1125 (11)	
H13A	0.3859	0.0498	1.0962	0.169*	
H13B	0.2872	0.0772	1.1523	0.169*	
H13C	0.3714	0.1016	1.0846	0.169*	
C14	0.3487 (2)	0.03179 (7)	0.92491 (14)	0.0623 (6)	
H14A	0.3778	0.0362	0.8690	0.075*	
H14B	0.4295	0.0347	0.9599	0.075*	
C15	0.2938 (3)	−0.01542 (8)	0.93300 (19)	0.0882 (8)	
H15A	0.2752	−0.0216	0.9894	0.132*	
H15B	0.3609	−0.0363	0.9134	0.132*	
H15C	0.2105	−0.0183	0.9013	0.132*	
C16	0.1177 (2)	0.06410 (7)	0.89560 (13)	0.0510 (5)	
C17	−0.0124 (2)	0.05148 (8)	0.91724 (15)	0.0670 (6)	
H17	−0.0425	0.0468	0.9703	0.080*	
C18	−0.0914 (2)	0.04693 (8)	0.84687 (15)	0.0714 (7)	
H18	−0.1831	0.0382	0.8440	0.086*	
C19	−0.0099 (2)	0.05753 (7)	0.78224 (14)	0.0560 (5)	
C20	0.0129 (2)	0.06853 (7)	0.57006 (14)	0.0581 (5)	
C21	−0.1045 (2)	0.04836 (8)	0.53895 (15)	0.0677 (6)	
H21	−0.1724	0.0386	0.5743	0.081*	
C22	−0.1214 (3)	0.04263 (9)	0.45690 (18)	0.0817 (8)	
H22	−0.2000	0.0289	0.4359	0.098*	
C23	−0.0225 (4)	0.05717 (9)	0.40677 (17)	0.0852 (8)	
C24	0.0946 (3)	0.07763 (10)	0.43399 (18)	0.0908 (8)	
H24	0.1604	0.0877	0.3977	0.109*	
C25	0.1123 (3)	0.08293 (9)	0.51780 (16)	0.0779 (7)	
H25	0.1919	0.0963	0.5384	0.093*	
C26	0.5873 (2)	0.03486 (8)	0.70026 (14)	0.0643 (6)	
H26	0.6421	0.0604	0.7003	0.077*	
C27	0.6470 (2)	−0.00680 (8)	0.69831 (15)	0.0713 (7)	
H27	0.7421	−0.0098	0.6979	0.086*	
C28	0.5647 (3)	−0.04364 (8)	0.69702 (14)	0.0666 (6)	
C29	0.4252 (3)	−0.04087 (8)	0.69818 (15)	0.0711 (6)	
H29	0.3713	−0.0666	0.6966	0.085*	
C30	0.3658 (2)	0.00074 (8)	0.70171 (14)	0.0645 (6)	
H30	0.2706	0.0033	0.7038	0.077*	
C31	0.4457 (2)	0.03883 (7)	0.70218 (12)	0.0540 (5)	
C32	0.3720 (2)	0.15340 (8)	0.70155 (14)	0.0598 (6)	
C33	0.4231 (3)	0.19583 (9)	0.70724 (18)	0.0832 (8)	
H33	0.5155	0.2037	0.7111	0.100*	
C34	0.3133 (3)	0.22504 (9)	0.70625 (18)	0.0804 (8)	
H34	0.3189	0.2560	0.7105	0.097*	
C35	0.1948 (2)	0.20081 (7)	0.69800 (14)	0.0590 (6)	
C36	0.0484 (2)	0.21568 (8)	0.68207 (14)	0.0638 (6)	
C37	0.0144 (3)	0.20811 (10)	0.59074 (16)	0.0842 (8)	
H37A	0.0156	0.1762	0.5801	0.101*	
H37B	−0.0785	0.2186	0.5800	0.101*	
C38	0.1100 (3)	0.23081 (13)	0.53122 (19)	0.1181 (12)	
H38A	0.1026	0.2627	0.5370	0.177*	
H38B	0.0851	0.2224	0.4764	0.177*	
H38C	0.2030	0.2217	0.5426	0.177*	
C39	0.0365 (3)	0.26561 (8)	0.70527 (19)	0.0865 (8)	
H39A	0.0651	0.2691	0.7619	0.104*	
H39B	0.0999	0.2826	0.6721	0.104*	
C40	−0.1071 (3)	0.28544 (11)	0.6945 (3)	0.1301 (13)	
H40A	−0.1360	0.2826	0.6384	0.195*	
H40B	−0.1054	0.3165	0.7095	0.195*	
H40C	−0.1702	0.2697	0.7287	0.195*	
C41	−0.0508 (2)	0.18925 (7)	0.73263 (15)	0.0602 (6)	
C42	−0.1721 (2)	0.16840 (9)	0.71117 (17)	0.0740 (7)	
H42	−0.2067	0.1649	0.6583	0.089*	
C43	−0.2337 (2)	0.15351 (9)	0.78168 (18)	0.0776 (7)	
H43	−0.3174	0.1386	0.7848	0.093*	
C44	−0.1493 (2)	0.16470 (8)	0.84609 (16)	0.0641 (6)	
C45	−0.0971 (3)	0.15716 (9)	1.05748 (18)	0.0792 (7)	
C46	0.0063 (4)	0.16878 (11)	1.1111 (2)	0.1044 (10)	
H46	0.0875	0.1810	1.0916	0.125*	
C47	−0.0098 (6)	0.16241 (15)	1.1938 (2)	0.1393 (16)	
H47	0.0604	0.1698	1.2305	0.167*	
C48	−0.1305 (9)	0.14515 (16)	1.2206 (3)	0.154 (3)	
C49	−0.2334 (6)	0.13401 (14)	1.1681 (3)	0.152 (2)	
H49	−0.3152	0.1225	1.1881	0.183*	
C50	−0.2186 (4)	0.13943 (10)	1.0868 (2)	0.1074 (11)	
H50	−0.2891	0.1313	1.0508	0.129*	

### Atomic displacement parameters (Å^2^)

**Table:**

	*U*^11^	*U*^22^	*U*^33^	*U*^12^	*U*^13^	*U*^23^
F1	0.189 (2)	0.0686 (11)	0.1451 (17)	0.0280 (12)	0.0206 (14)	−0.0108 (10)
F2	0.1882 (19)	0.1245 (14)	0.0651 (11)	0.0197 (13)	−0.0169 (11)	−0.0180 (9)
F3	0.1163 (12)	0.0668 (9)	0.0986 (12)	0.0183 (8)	−0.0075 (9)	−0.0041 (8)
F4	0.431 (5)	0.162 (2)	0.0912 (17)	0.094 (3)	0.086 (2)	0.0330 (14)
N1	0.0591 (12)	0.0611 (12)	0.0887 (15)	−0.0049 (9)	0.0045 (10)	−0.0075 (10)
N2	0.0555 (12)	0.0674 (13)	0.0771 (14)	−0.0066 (10)	−0.0020 (9)	−0.0066 (10)
N3	0.0424 (9)	0.0630 (11)	0.0621 (11)	−0.0021 (9)	−0.0036 (8)	−0.0002 (9)
N4	0.0407 (9)	0.0577 (10)	0.0531 (11)	−0.0055 (8)	−0.0019 (7)	0.0068 (8)
N5	0.0469 (10)	0.0633 (11)	0.0633 (12)	−0.0065 (9)	−0.0072 (9)	0.0061 (9)
N6	0.0499 (10)	0.0599 (11)	0.0608 (12)	−0.0035 (8)	−0.0084 (9)	0.0030 (9)
N7	0.0456 (10)	0.0608 (11)	0.0614 (11)	−0.0048 (9)	−0.0045 (8)	−0.0004 (9)
N8	0.0472 (10)	0.0640 (12)	0.0712 (12)	−0.0051 (9)	−0.0042 (8)	0.0056 (9)
N9	0.0442 (9)	0.0545 (11)	0.0662 (11)	−0.0051 (8)	−0.0060 (8)	0.0075 (8)
N10	0.0445 (10)	0.0611 (11)	0.0701 (13)	−0.0022 (8)	−0.0070 (8)	0.0043 (9)
N11	0.0510 (11)	0.0714 (13)	0.0917 (16)	0.0023 (10)	0.0079 (11)	0.0086 (11)
N12	0.0609 (12)	0.0704 (13)	0.0751 (14)	0.0035 (10)	0.0075 (10)	0.0043 (10)
C1	0.080 (2)	0.0691 (19)	0.214 (4)	−0.0035 (16)	0.036 (2)	−0.023 (2)
C2	0.100 (2)	0.076 (2)	0.219 (5)	0.0085 (19)	0.052 (3)	−0.012 (2)
C3	0.126 (3)	0.0629 (19)	0.092 (2)	0.0145 (19)	0.0153 (19)	−0.0024 (15)
C4	0.122 (3)	0.0551 (18)	0.133 (3)	−0.0151 (17)	−0.026 (2)	−0.0022 (16)
C5	0.0820 (18)	0.0678 (18)	0.106 (2)	−0.0094 (14)	−0.0132 (15)	0.0040 (15)
C6	0.0688 (15)	0.0578 (14)	0.0745 (16)	−0.0044 (12)	0.0057 (12)	−0.0019 (11)
C7	0.0494 (12)	0.0625 (15)	0.0708 (15)	−0.0060 (11)	−0.0063 (10)	−0.0076 (11)
C8	0.0479 (13)	0.0799 (18)	0.100 (2)	−0.0111 (12)	−0.0013 (12)	−0.0115 (14)
C9	0.0485 (13)	0.0728 (17)	0.0853 (18)	0.0025 (12)	−0.0042 (11)	−0.0091 (13)
C10	0.0447 (12)	0.0650 (14)	0.0524 (13)	0.0030 (10)	−0.0085 (9)	−0.0034 (10)
C11	0.0496 (11)	0.0603 (13)	0.0500 (12)	0.0026 (10)	−0.0034 (9)	0.0033 (10)
C12	0.0743 (15)	0.0819 (16)	0.0540 (14)	0.0089 (13)	0.0002 (11)	0.0065 (12)
C13	0.107 (2)	0.175 (3)	0.0549 (17)	0.010 (2)	−0.0159 (15)	0.0015 (18)
C14	0.0538 (12)	0.0678 (15)	0.0651 (14)	0.0081 (11)	−0.0062 (10)	0.0024 (11)
C15	0.0784 (17)	0.0677 (17)	0.119 (2)	0.0130 (14)	0.0034 (16)	0.0059 (15)
C16	0.0467 (11)	0.0525 (12)	0.0538 (13)	0.0009 (9)	−0.0001 (9)	0.0051 (9)
C17	0.0537 (13)	0.0874 (17)	0.0602 (15)	−0.0058 (12)	0.0070 (11)	0.0100 (12)
C18	0.0474 (12)	0.0902 (18)	0.0766 (17)	−0.0153 (12)	−0.0019 (12)	0.0120 (13)
C19	0.0450 (11)	0.0587 (13)	0.0640 (15)	−0.0065 (10)	−0.0082 (10)	0.0044 (10)
C20	0.0590 (13)	0.0545 (13)	0.0605 (14)	0.0020 (10)	−0.0092 (11)	0.0017 (10)
C21	0.0640 (14)	0.0700 (15)	0.0686 (16)	0.0050 (12)	−0.0167 (12)	0.0005 (12)
C22	0.0863 (19)	0.0810 (18)	0.0769 (19)	0.0105 (15)	−0.0258 (16)	−0.0067 (14)
C23	0.123 (3)	0.0718 (17)	0.0605 (18)	0.0161 (17)	−0.0222 (17)	−0.0089 (13)
C24	0.120 (2)	0.0840 (19)	0.0690 (19)	−0.0030 (18)	0.0186 (17)	−0.0007 (14)
C25	0.0829 (17)	0.0791 (17)	0.0715 (18)	−0.0124 (14)	0.0008 (14)	−0.0032 (13)
C26	0.0518 (13)	0.0643 (15)	0.0769 (16)	−0.0034 (11)	−0.0011 (11)	0.0005 (12)
C27	0.0578 (14)	0.0747 (17)	0.0813 (18)	0.0076 (13)	−0.0045 (12)	−0.0028 (13)
C28	0.0803 (17)	0.0614 (15)	0.0580 (14)	0.0077 (13)	−0.0056 (12)	−0.0028 (11)
C29	0.0800 (17)	0.0631 (15)	0.0700 (16)	−0.0157 (13)	−0.0029 (13)	−0.0047 (12)
C30	0.0520 (13)	0.0711 (16)	0.0704 (15)	−0.0099 (12)	−0.0031 (11)	−0.0082 (12)
C31	0.0500 (12)	0.0616 (13)	0.0503 (12)	−0.0036 (10)	−0.0039 (9)	−0.0005 (10)
C32	0.0440 (12)	0.0618 (14)	0.0735 (15)	−0.0051 (11)	−0.0055 (10)	0.0077 (11)
C33	0.0543 (14)	0.0709 (17)	0.124 (2)	−0.0132 (13)	−0.0100 (14)	0.0101 (15)
C34	0.0686 (16)	0.0564 (14)	0.116 (2)	−0.0098 (13)	−0.0123 (14)	0.0081 (14)
C35	0.0569 (13)	0.0539 (13)	0.0661 (15)	−0.0009 (11)	−0.0040 (10)	0.0089 (10)
C36	0.0580 (13)	0.0621 (14)	0.0713 (16)	0.0078 (11)	−0.0036 (11)	0.0096 (11)
C37	0.0736 (16)	0.104 (2)	0.0743 (18)	0.0214 (15)	−0.0102 (13)	0.0177 (15)
C38	0.111 (2)	0.164 (3)	0.080 (2)	0.024 (2)	0.0075 (18)	0.037 (2)
C39	0.0858 (18)	0.0601 (16)	0.114 (2)	0.0114 (13)	0.0128 (16)	0.0166 (14)
C40	0.110 (3)	0.088 (2)	0.193 (4)	0.041 (2)	0.009 (2)	0.015 (2)
C41	0.0493 (12)	0.0600 (13)	0.0711 (16)	0.0097 (10)	−0.0072 (11)	0.0038 (11)
C42	0.0562 (14)	0.0828 (17)	0.0824 (18)	−0.0011 (13)	−0.0166 (13)	−0.0020 (14)
C43	0.0465 (13)	0.0840 (18)	0.102 (2)	−0.0086 (12)	−0.0118 (13)	0.0026 (15)
C44	0.0428 (12)	0.0669 (15)	0.0826 (18)	−0.0025 (11)	0.0031 (11)	0.0069 (12)
C45	0.095 (2)	0.0657 (16)	0.0775 (19)	0.0211 (14)	0.0204 (16)	0.0099 (13)
C46	0.118 (3)	0.121 (3)	0.074 (2)	0.032 (2)	0.0074 (18)	−0.0031 (18)
C47	0.202 (5)	0.142 (4)	0.074 (3)	0.075 (3)	0.005 (3)	−0.005 (2)
C48	0.290 (8)	0.105 (3)	0.070 (3)	0.080 (4)	0.065 (4)	0.025 (2)
C49	0.238 (6)	0.102 (3)	0.118 (4)	0.018 (3)	0.088 (4)	0.034 (3)
C50	0.135 (3)	0.081 (2)	0.107 (3)	−0.0010 (19)	0.046 (2)	0.0179 (17)

### Geometric parameters (Å, °)

**Table:**

F1—C3	1.358 (3)	C17—C18	1.383 (3)
F2—C23	1.361 (3)	C17—H17	0.9300
F3—C28	1.353 (3)	C18—C19	1.367 (3)
F4—C48	1.340 (4)	C18—H18	0.9300
N1—N2	1.267 (2)	C20—C25	1.369 (3)
N1—C6	1.416 (3)	C20—C21	1.383 (3)
N2—C7	1.365 (3)	C21—C22	1.362 (3)
N3—C10	1.360 (3)	C21—H21	0.9300
N3—C7	1.366 (3)	C22—C23	1.345 (4)
N3—H3	0.8600	C22—H22	0.9300
N4—C16	1.359 (2)	C23—C24	1.362 (4)
N4—C19	1.374 (2)	C24—C25	1.389 (4)
N4—H4	0.8600	C24—H24	0.9300
N5—N6	1.266 (2)	C25—H25	0.9300
N5—C19	1.366 (3)	C26—C27	1.375 (3)
N6—C20	1.418 (3)	C26—C31	1.383 (3)
N7—N8	1.262 (2)	C26—H26	0.9300
N7—C31	1.417 (3)	C27—C28	1.361 (3)
N8—C32	1.367 (3)	C27—H27	0.9300
N9—C35	1.359 (3)	C28—C29	1.360 (3)
N9—C32	1.375 (3)	C29—C30	1.373 (3)
N9—H9	0.8600	C29—H29	0.9300
N10—C41	1.359 (3)	C30—C31	1.379 (3)
N10—C44	1.369 (3)	C30—H30	0.9300
N10—H10	0.8600	C32—C33	1.365 (3)
N11—N12	1.269 (3)	C33—C34	1.380 (3)
N11—C44	1.367 (3)	C33—H33	0.9300
N12—C45	1.410 (3)	C34—C35	1.366 (3)
C1—C2	1.370 (4)	C34—H34	0.9300
C1—C6	1.372 (4)	C35—C36	1.512 (3)
C1—H1	0.9300	C36—C41	1.503 (3)
C2—C3	1.330 (4)	C36—C37	1.543 (3)
C2—H2	0.9300	C36—C39	1.545 (3)
C3—C4	1.339 (4)	C37—C38	1.516 (4)
C4—C5	1.386 (4)	C37—H37A	0.9700
C4—H4A	0.9300	C37—H37B	0.9700
C5—C6	1.360 (3)	C38—H38A	0.9600
C5—H5	0.9300	C38—H38B	0.9600
C7—C8	1.375 (3)	C38—H38C	0.9600
C8—C9	1.381 (3)	C39—C40	1.526 (4)
C8—H8	0.9300	C39—H39A	0.9700
C9—C10	1.377 (3)	C39—H39B	0.9700
C9—H9A	0.9300	C40—H40A	0.9600
C10—C11	1.502 (3)	C40—H40B	0.9600
C11—C16	1.507 (3)	C40—H40C	0.9600
C11—C14	1.542 (3)	C41—C42	1.376 (3)
C11—C12	1.545 (3)	C42—C43	1.381 (4)
C12—C13	1.514 (4)	C42—H42	0.9300
C12—H12A	0.9700	C43—C44	1.369 (3)
C12—H12B	0.9700	C43—H43	0.9300
C13—H13A	0.9600	C45—C46	1.371 (4)
C13—H13B	0.9600	C45—C50	1.386 (4)
C13—H13C	0.9600	C46—C47	1.378 (5)
C14—C15	1.516 (3)	C46—H46	0.9300
C14—H14A	0.9700	C47—C48	1.361 (7)
C14—H14B	0.9700	C47—H47	0.9300
C15—H15A	0.9600	C48—C49	1.353 (7)
C15—H15B	0.9600	C49—C50	1.350 (5)
C15—H15C	0.9600	C49—H49	0.9300
C16—C17	1.372 (3)	C50—H50	0.9300
			
N2—N1—C6	114.72 (19)	C21—C22—H22	120.6
N1—N2—C7	113.93 (19)	C22—C23—F2	120.0 (3)
C10—N3—C7	109.63 (17)	C22—C23—C24	123.2 (3)
C10—N3—H3	125.2	F2—C23—C24	116.8 (3)
C7—N3—H3	125.2	C23—C24—C25	117.8 (3)
C16—N4—C19	109.18 (17)	C23—C24—H24	121.1
C16—N4—H4	125.4	C25—C24—H24	121.1
C19—N4—H4	125.4	C20—C25—C24	120.1 (3)
N6—N5—C19	114.43 (17)	C20—C25—H25	120.0
N5—N6—C20	114.22 (17)	C24—C25—H25	120.0
N8—N7—C31	114.70 (17)	C27—C26—C31	119.9 (2)
N7—N8—C32	114.22 (17)	C27—C26—H26	120.0
C35—N9—C32	109.45 (18)	C31—C26—H26	120.0
C35—N9—H9	125.3	C28—C27—C26	119.0 (2)
C32—N9—H9	125.3	C28—C27—H27	120.5
C41—N10—C44	109.28 (18)	C26—C27—H27	120.5
C41—N10—H10	125.4	F3—C28—C29	118.7 (2)
C44—N10—H10	125.4	F3—C28—C27	118.8 (2)
N12—N11—C44	114.79 (19)	C29—C28—C27	122.5 (2)
N11—N12—C45	114.0 (2)	C28—C29—C30	118.4 (2)
C2—C1—C6	121.7 (3)	C28—C29—H29	120.8
C2—C1—H1	119.2	C30—C29—H29	120.8
C6—C1—H1	119.2	C29—C30—C31	120.7 (2)
C3—C2—C1	118.5 (3)	C29—C30—H30	119.6
C3—C2—H2	120.8	C31—C30—H30	119.6
C1—C2—H2	120.8	C30—C31—C26	119.4 (2)
C2—C3—C4	122.1 (3)	C30—C31—N7	115.08 (18)
C2—C3—F1	119.5 (3)	C26—C31—N7	125.53 (19)
C4—C3—F1	118.4 (3)	C33—C32—N8	127.3 (2)
C3—C4—C5	119.9 (3)	C33—C32—N9	107.1 (2)
C3—C4—H4A	120.1	N8—C32—N9	125.62 (19)
C5—C4—H4A	120.1	C32—C33—C34	107.8 (2)
C6—C5—C4	119.6 (3)	C32—C33—H33	126.1
C6—C5—H5	120.2	C34—C33—H33	126.1
C4—C5—H5	120.2	C35—C34—C33	108.5 (2)
C5—C6—C1	118.3 (2)	C35—C34—H34	125.7
C5—C6—N1	125.2 (2)	C33—C34—H34	125.7
C1—C6—N1	116.5 (2)	N9—C35—C34	107.08 (19)
N2—C7—N3	125.2 (2)	N9—C35—C36	121.68 (19)
N2—C7—C8	127.3 (2)	C34—C35—C36	130.8 (2)
N3—C7—C8	107.4 (2)	C41—C36—C35	111.12 (17)
C7—C8—C9	107.7 (2)	C41—C36—C37	109.0 (2)
C7—C8—H8	126.2	C35—C36—C37	108.28 (19)
C9—C8—H8	126.2	C41—C36—C39	108.8 (2)
C10—C9—C8	108.2 (2)	C35—C36—C39	108.3 (2)
C10—C9—H9A	125.9	C37—C36—C39	111.3 (2)
C8—C9—H9A	125.9	C38—C37—C36	115.5 (2)
N3—C10—C9	107.1 (2)	C38—C37—H37A	108.4
N3—C10—C11	121.60 (17)	C36—C37—H37A	108.4
C9—C10—C11	130.9 (2)	C38—C37—H37B	108.4
C10—C11—C16	110.40 (16)	C36—C37—H37B	108.4
C10—C11—C14	108.90 (17)	H37A—C37—H37B	107.5
C16—C11—C14	109.26 (17)	C37—C38—H38A	109.5
C10—C11—C12	108.74 (18)	C37—C38—H38B	109.5
C16—C11—C12	108.19 (17)	H38A—C38—H38B	109.5
C14—C11—C12	111.35 (17)	C37—C38—H38C	109.5
C13—C12—C11	115.4 (2)	H38A—C38—H38C	109.5
C13—C12—H12A	108.4	H38B—C38—H38C	109.5
C11—C12—H12A	108.4	C40—C39—C36	114.7 (2)
C13—C12—H12B	108.4	C40—C39—H39A	108.6
C11—C12—H12B	108.4	C36—C39—H39A	108.6
H12A—C12—H12B	107.5	C40—C39—H39B	108.6
C12—C13—H13A	109.5	C36—C39—H39B	108.6
C12—C13—H13B	109.5	H39A—C39—H39B	107.6
H13A—C13—H13B	109.5	C39—C40—H40A	109.5
C12—C13—H13C	109.5	C39—C40—H40B	109.5
H13A—C13—H13C	109.5	H40A—C40—H40B	109.5
H13B—C13—H13C	109.5	C39—C40—H40C	109.5
C15—C14—C11	115.39 (19)	H40A—C40—H40C	109.5
C15—C14—H14A	108.4	H40B—C40—H40C	109.5
C11—C14—H14A	108.4	N10—C41—C42	107.2 (2)
C15—C14—H14B	108.4	N10—C41—C36	121.78 (19)
C11—C14—H14B	108.4	C42—C41—C36	130.7 (2)
H14A—C14—H14B	107.5	C41—C42—C43	108.2 (2)
C14—C15—H15A	109.5	C41—C42—H42	125.9
C14—C15—H15B	109.5	C43—C42—H42	125.9
H15A—C15—H15B	109.5	C44—C43—C42	107.6 (2)
C14—C15—H15C	109.5	C44—C43—H43	126.2
H15A—C15—H15C	109.5	C42—C43—H43	126.2
H15B—C15—H15C	109.5	N11—C44—C43	126.6 (2)
N4—C16—C17	107.24 (18)	N11—C44—N10	125.7 (2)
N4—C16—C11	122.05 (17)	C43—C44—N10	107.7 (2)
C17—C16—C11	130.5 (2)	C46—C45—C50	119.8 (3)
C16—C17—C18	108.5 (2)	C46—C45—N12	115.9 (3)
C16—C17—H17	125.8	C50—C45—N12	124.2 (3)
C18—C17—H17	125.8	C45—C46—C47	120.1 (4)
C19—C18—C17	107.5 (2)	C45—C46—H46	119.9
C19—C18—H18	126.3	C47—C46—H46	119.9
C17—C18—H18	126.3	C48—C47—C46	118.6 (5)
N5—C19—C18	127.26 (19)	C48—C47—H47	120.7
N5—C19—N4	125.11 (19)	C46—C47—H47	120.7
C18—C19—N4	107.62 (19)	F4—C48—C49	120.3 (7)
C25—C20—C21	119.6 (2)	F4—C48—C47	118.2 (7)
C25—C20—N6	115.7 (2)	C49—C48—C47	121.5 (4)
C21—C20—N6	124.6 (2)	C50—C49—C48	120.6 (5)
C22—C21—C20	120.4 (3)	C50—C49—H49	119.7
C22—C21—H21	119.8	C48—C49—H49	119.7
C20—C21—H21	119.8	C49—C50—C45	119.3 (4)
C23—C22—C21	118.9 (3)	C49—C50—H50	120.4
C23—C22—H22	120.6	C45—C50—H50	120.4
			
C6—N1—N2—C7	173.2 (2)	N6—C20—C25—C24	177.1 (2)
C19—N5—N6—C20	173.69 (17)	C23—C24—C25—C20	−1.1 (4)
C31—N7—N8—C32	177.07 (18)	C31—C26—C27—C28	1.0 (4)
C44—N11—N12—C45	177.15 (19)	C26—C27—C28—F3	180.0 (2)
C6—C1—C2—C3	0.0 (6)	C26—C27—C28—C29	−0.5 (4)
C1—C2—C3—C4	1.4 (6)	F3—C28—C29—C30	178.8 (2)
C1—C2—C3—F1	−179.6 (3)	C27—C28—C29—C30	−0.7 (4)
C2—C3—C4—C5	−1.1 (5)	C28—C29—C30—C31	1.5 (3)
F1—C3—C4—C5	179.9 (3)	C29—C30—C31—C26	−0.9 (3)
C3—C4—C5—C6	−0.6 (5)	C29—C30—C31—N7	−179.8 (2)
C4—C5—C6—C1	1.9 (4)	C27—C26—C31—C30	−0.3 (3)
C4—C5—C6—N1	−177.6 (3)	C27—C26—C31—N7	178.5 (2)
C2—C1—C6—C5	−1.6 (5)	N8—N7—C31—C30	−175.84 (19)
C2—C1—C6—N1	177.9 (3)	N8—N7—C31—C26	5.3 (3)
N2—N1—C6—C5	3.5 (4)	N7—N8—C32—C33	171.3 (2)
N2—N1—C6—C1	−176.0 (3)	N7—N8—C32—N9	−8.6 (3)
N1—N2—C7—N3	−11.0 (3)	C35—N9—C32—C33	0.0 (3)
N1—N2—C7—C8	172.0 (2)	C35—N9—C32—N8	179.9 (2)
C10—N3—C7—N2	−176.8 (2)	N8—C32—C33—C34	−179.0 (2)
C10—N3—C7—C8	0.8 (2)	N9—C32—C33—C34	0.9 (3)
N2—C7—C8—C9	177.6 (2)	C32—C33—C34—C35	−1.4 (3)
N3—C7—C8—C9	0.1 (3)	C32—N9—C35—C34	−0.9 (3)
C7—C8—C9—C10	−0.9 (3)	C32—N9—C35—C36	172.0 (2)
C7—N3—C10—C9	−1.3 (2)	C33—C34—C35—N9	1.4 (3)
C7—N3—C10—C11	172.35 (18)	C33—C34—C35—C36	−170.6 (2)
C8—C9—C10—N3	1.4 (3)	N9—C35—C36—C41	49.7 (3)
C8—C9—C10—C11	−171.5 (2)	C34—C35—C36—C41	−139.3 (3)
N3—C10—C11—C16	51.0 (2)	N9—C35—C36—C37	−69.9 (3)
C9—C10—C11—C16	−137.0 (2)	C34—C35—C36—C37	101.1 (3)
N3—C10—C11—C14	170.98 (18)	N9—C35—C36—C39	169.2 (2)
C9—C10—C11—C14	−17.0 (3)	C34—C35—C36—C39	−19.8 (4)
N3—C10—C11—C12	−67.5 (2)	C41—C36—C37—C38	−177.1 (2)
C9—C10—C11—C12	104.5 (3)	C35—C36—C37—C38	−56.1 (3)
C10—C11—C12—C13	−56.6 (3)	C39—C36—C37—C38	62.9 (3)
C16—C11—C12—C13	−176.6 (2)	C41—C36—C39—C40	−57.7 (3)
C14—C11—C12—C13	63.4 (3)	C35—C36—C39—C40	−178.6 (2)
C10—C11—C14—C15	−176.32 (19)	C37—C36—C39—C40	62.4 (3)
C16—C11—C14—C15	−55.7 (3)	C44—N10—C41—C42	−1.0 (2)
C12—C11—C14—C15	63.8 (3)	C44—N10—C41—C36	173.21 (19)
C19—N4—C16—C17	−1.5 (2)	C35—C36—C41—N10	54.7 (3)
C19—N4—C16—C11	173.95 (18)	C37—C36—C41—N10	173.91 (19)
C10—C11—C16—N4	55.3 (2)	C39—C36—C41—N10	−64.5 (3)
C14—C11—C16—N4	−64.4 (2)	C35—C36—C41—C42	−132.6 (3)
C12—C11—C16—N4	174.20 (18)	C37—C36—C41—C42	−13.4 (3)
C10—C11—C16—C17	−130.4 (2)	C39—C36—C41—C42	108.2 (3)
C14—C11—C16—C17	109.9 (3)	N10—C41—C42—C43	1.1 (3)
C12—C11—C16—C17	−11.5 (3)	C36—C41—C42—C43	−172.4 (2)
N4—C16—C17—C18	1.6 (3)	C41—C42—C43—C44	−0.8 (3)
C11—C16—C17—C18	−173.4 (2)	N12—N11—C44—C43	171.1 (2)
C16—C17—C18—C19	−1.0 (3)	N12—N11—C44—N10	−8.8 (3)
N6—N5—C19—C18	171.6 (2)	C42—C43—C44—N11	−179.7 (2)
N6—N5—C19—N4	−9.8 (3)	C42—C43—C44—N10	0.2 (3)
C17—C18—C19—N5	178.9 (2)	C41—N10—C44—N11	−179.6 (2)
C17—C18—C19—N4	0.1 (3)	C41—N10—C44—C43	0.5 (3)
C16—N4—C19—N5	−177.96 (19)	N11—N12—C45—C46	−175.6 (2)
C16—N4—C19—C18	0.9 (2)	N11—N12—C45—C50	4.6 (4)
N5—N6—C20—C25	−175.0 (2)	C50—C45—C46—C47	0.7 (4)
N5—N6—C20—C21	1.4 (3)	N12—C45—C46—C47	−179.1 (3)
C25—C20—C21—C22	0.3 (3)	C45—C46—C47—C48	−1.0 (5)
N6—C20—C21—C22	−176.0 (2)	C46—C47—C48—F4	−178.9 (3)
C20—C21—C22—C23	−0.4 (4)	C46—C47—C48—C49	0.4 (7)
C21—C22—C23—F2	178.7 (2)	F4—C48—C49—C50	179.9 (3)
C21—C22—C23—C24	−0.2 (4)	C47—C48—C49—C50	0.5 (7)
C22—C23—C24—C25	1.0 (4)	C48—C49—C50—C45	−0.9 (6)
F2—C23—C24—C25	−177.9 (2)	C46—C45—C50—C49	0.3 (5)
C21—C20—C25—C24	0.5 (4)	N12—C45—C50—C49	−179.9 (3)

### Hydrogen-bond geometry (Å, °)

**Table:**

*D*—H···*A*	*D*—H	H···*A*	*D*···*A*	*D*—H···*A*
N3—H3···N12	0.86	2.27	3.121 (3)	171
N4—H4···N7	0.86	2.22	3.062 (2)	166
N9—H9···N6	0.86	2.31	3.160 (3)	168
N10—H10···N1	0.86	2.26	3.092 (3)	163
